# Ameliorated reserpine production via in vitro direct and indirect regeneration system in *Rauvolfia serpentina* (L.) Benth. ex Kurz.

**DOI:** 10.1007/s13205-020-02285-3

**Published:** 2020-06-08

**Authors:** Eashan Mukherjee, Sutanu Sarkar, Somnath Bhattacharyya, Saikat Gantait

**Affiliations:** Crop Research Unit (Genetics and Plant Breeding), Bidhan Chandra Krishi Viswavidyalaya, Mohanpur, Nadia, West Bengal 741252 India

**Keywords:** Biomass, Callus, HPTLC, Multiple shoots, Plant growth regulator, Secondary metabolite

## Abstract

*Rauvolfia serpentina* (L.) Benth. ex Kurz., popularly known as Indian Snakeroot plant, belonging to Apocynaceae family, holds immense medicinal importance, owing to its rich source of multiple secondary metabolites such as ajmaline, ajmalicine, reserpine, and serpentine. To meet the constant demands for the key secondary metabolite (reserpine) by majority of the pharmaceutical industries, the present study assessed the effects of direct and indirect regeneration system on amelioration of reserpine accumulation in shoots of *R. serpentina*. In vitro multiple shoot cultures were established using shoot tip explants. Best results for shoot initiation, multiplication, and biomass production were obtained in case of Murashige and Skoog medium, supplemented with 1 mg/l *N*^6^-benzyladenine. The multiple shoots were then sub-cultured on cytokinin–auxin combination media for further proliferation. Highest shoot and leaf multiplication rates and the most enhanced biomass were obtained in case of 1–1.5 mg/l Kinetin + 0.2 mg/l α-naphthalene acetic acid (NAA). Callus induction and its subsequent proliferation was obtained using 1.5 mg/l 2,4-dichlorophenoxyacetic acid. The best indirect shoot regeneration with highest shoot and leaf proliferation from calli was observed in case of 1 mg/l thidiazuron + 0.2 mg/l NAA. Reserpine content estimation via HPTLC from in vitro shoots (direct regeneration) and calli (indirect regeneration) were recorded to undergo an almost three-fold and two-fold increment (respectively) in comparison to that of the mother plant. Thus, in vitro direct regeneration system proved to be more effective and efficient in ameliorating the reserpine content.

## Introduction

*Rauvolfia* genus comprises of different species of plants which have diverse medicinal and allied uses. The genus belongs to the family Apocynaceae and order Gentianales, under the kingdom Plantae. These plants are erect, evergreen, perennial shrubs growing up to a height of 60–90 cm (Mukherjee et al. 2019). Amongst different species, *Rauvolfia serpentina* (L.) Benth. ex Kurz. finds itself to be quite indispensable owing to the presence of assorted secondary metabolites, which have varied medicinal and therapeutic uses. Centre of origin of the plant is South-East Asia. The plant is extensively distributed in the tropical zones of America, Africa, India, Sri Lanka, Myanmar, and Nepal. In India, the distribution covers parts of tropical Himalaya, Gangetic plains, sub-Himalayan territories covering Shimla to Assam, and Sikkim to Nepal and Bhutan. Almost all parts of the plant such as leaves, shoots, flowers, and especially the roots contain several secondary metabolites like ajmaline, ajmalicine, rescinamine, reserpine, serpentine, etc. These compounds are mainly indole alkaloids that are biosynthesised from the aromatic acid tryptophan. Since ancient times, this plant has been utilized in Ayurveda and Unani as remedies for fever, hypertension, insomnia, epilepsy, psychosis, schizophrenia, and other central nervous system disorders (reviewed by Mukherjee et al. 2019). Root extracts have been used as a cure for snakebites and ulcers (Lobay 2015; Bunkar 2017). As per the reports of Soni et al. (2016), root bark find its usage as a hypnotic and sedative which aids in lowering down blood pressure.

By and large, this plant is propagated via seeds; stem cuttings and root cuttings have been reported in some cases as well (Ghate et al. 2019). However, these traditional ways of propagation are subject to several limitations, viz., low seed set, poor rooting (Khan et al. 2018), and high occurrences of genetic variation in seed propagated populations (Pillai et al. 2012). Lucrative pharmacological and ethnobotanical uses (due the presence of the above-mentioned secondary metabolites) have led to the exploitation of *R. serpentina* natural resources, thereby pushing the species to a threatened category as per IUCN. Keeping in view these constraints that are involved with the propagation and conservation of this plant species; in vitro tissue culture technologies offer a practical and constructive solution to the preservation and maintenance of this plant. It is hypothesized that in vitro propagation technology of *R. serpentina*, following direct and indirect (callus-mediated) regeneration for enhancement of biomass and secondary metabolites production, would be a significant contribution to the pharmacological industry at large, since both biomass-cum-secondary metabolite production would be facilitated simultaneously.

Arrays of reports are available on all these aspects of reserpine production via different in vitro biotechnological interventions, discretely (reviewed by Mukherjee et al. 2019). For instance, there are quite a few reports on the production of reserpine from in vitro plant cell, tissue, and organ cultures using transformed hairy roots (Pandey et al. 2014), elicitor-mediated shoot apex and nodal segments (Panwar and Guru 2015), and germinated synthetic seeds (Gantait and Kundu 2017), etc. However, till date, to the best of our knowledge, the inclusive reports are very limited, since none of them covers all of these aspects on *R. serpentina* in one singular comprehensive report. Based on this backdrop, the present study deals with the optimization of in vitro direct multiple shoot and indirect (callus-mediated) multiple shoot regeneration system in *R. serpentina* for high biomass production and eventually to ameliorate the reserpine level among in vitro regenerants.

## Materials and methods

### Explant collection and surface disinfection

Shoot apices (1–1.2 cm) were collected from the Medicinal Plant Garden, Bidhan Chandra Krishi Viswavidyalaya, Mohanpur, India. Surface disinfection of the shoot apex explants was done following a series of treatments using autoclaved (at 1.1 kg/cm^2^ pressure and 121 °C for 15 min) solutions of 0.03% (w/v) bavistin, 10% (v/v) Tween-20, 20% (v/v) sodium hypochlorite (NaOCl), 1% (w/v) cetrimide, ethyl alcohol (C_2_H_5_OH), and 0.1% (w/v) mercuric chloride (HgCl_2_) (all these were obtained from Merck Life Sciences. Pvt. Ltd., India), carried out in the Laminar Air Flow cabinet (LAF). All of the sterilants were used for 5 min with the exception of ethyl alcohol, which was used for 30 s. After disinfection, the explants were taken out and the exposed basal portions of the shoot apices were trimmed and removed.

### Preparation of media and culture conditions

The basal medium used for the experiments was the Murashige and Skoog (MS) (1962) semisolid medium and the same was prepared by adding ready MS salt (Himedia laboratories Pvt. Ltd, India) 4.4 g/l supplemented with 30 g/l sucrose (Merck life Sciences Pvt. Ltd., India), 100 mg/l m-inositol, and 7 g/l agar (both obtained from Sisco Research Laboratories Pvt. Ltd., India). For the different experiments on direct (shoot initiation and multiplication) and indirect (callus-mediated multiple shoot) regeneration, the media were supplemented with a choice and concentrations of plant growth regulators (PGRs). The pH of the media was adjusted to 5.7 by addition of 0.1 N HCl or 0.1 N NaOH following supplementation of PGRs but before addition of agar. The media were prepared in 55 ml culture tubes (Borosil, India) containing 20 ml media and were then sterilized using autoclave. For callus induction experiment, 55 ml culture tubes (each containing 10 ml of basal medium) were used, which were then tilted (before solidification, after autoclaving) at an angle of less than 45° (to increase the surface area for the explants inoculation). All the cultures were maintained at 25 ± 1 °C temperature under cool white fluorescent lights (Phillips Champion, PHILLIPS, India), 50 µmol/m^2^/s photosynthetic photon flux density, under 16 h photoperiod, and at 60% RH; for callus induction experiment, the culture tubes were initially kept under dark for 72 h and then transferred to 16 h photoperiod.

### Establishment of direct in vitro multiple shoot culture

For the establishment of the in vitro shoot culture, shoot apices were inoculated in MS medium supplemented with the following PGRs (at the rate of 0.5, 1, 1.5, 2, and 2.5 mg/l)—*N*^6^-benzyladenine (BA), Kinetin (KIN), thidiazuron (TDZ), zeatin (all obtained from Sisco Research Laboratories Pvt. Ltd., India), and meta-Topoline (mT) (Titan Biotech Ltd., India). Along with the above-mentioned PGRs, the shoot apices were also inoculated in PGR-free MS medium (served as the control). Data on response of explants to shoot induction (%) and days to fresh shoot induction were taken on a daily basis, whereas number of shoots, shoot length (mm), and number of leaves, along with fresh weight (mg) and dry weight (mg), were recorded at 42 days of culture. Dry weight was taken after drying the shoot clumps under hot-air oven for 24 h at 50 °C.

### Proliferation of in vitro multiple shoot culture

After final data collection, individual shoots (1–1.2 cm size) having one inter-nodal region were isolated and transferred in shoot proliferation media wherein PGRs were used in combinations [BA, KIN, and TDZ, each of them supplemented with α-naphthalene acetic acid (NAA) (Sisco Research Laboratories Pvt. Ltd., India)]. The concentrations of the PGRs were: 0.5, 1, 1.5 mg/l each of them (cytokinin) supplemented with 0.2 mg/l NAA. Along with the above-mentioned PGRs, the shoots were also inoculated in PGR-free MS medium (served as the control). Data on response of shoots towards proliferation (%), days to initiation of multiple shoot proliferation were taken on a daily basis, whereas number of shoots, shoot length (mm), and number of leaves, along with fresh weight (mg) and dry weight (mg), were recorded at 42 days of culture. Dry weight was taken after drying the shoot clumps under hot-air oven for 24 h at 50 °C.

### Establishment of in vitro callus culture

For the in vitro callus induction experiment, the preferred explant was leaf, which was obtained from the previously optimized multiple shoot culture experiment. Three PGRs (at concentrations of 0.5, 1, 1.5, and 2 mg/l), viz., 2,4-dichlorophenoxy acetic acid (2,4-D) (Loba Chemie Pvt. Ltd., India), BA, and KIN were supplemented with MS basal medium. Before inoculation, the leaves were wounded horizontally against the leaf midrib with the help of scalpel, thereby increasing the chances of callus induction from the wounded sites. Along with the above-mentioned PGRs, the leaves were also inoculated in PGR-free MS medium (served as the control). Data on response of explants towards callus induction (%) and days to induction of the calli were recorded on a daily basis, and finally, fresh weight (mg) and dry weight (mg) of the calli were taken after 56 days of culture. Dry weight was taken after drying the calli under hot-air oven for 24 h at 50 °C.

### Establishment of in vitro indirect shoot regeneration from calli

Organogenic calli obtained from the callus induction experiment involving the treatments of 1–1.5 mg/l 2,4-D were used for indirect shoot regeneration purpose. Isolated calli (each weighing 300 mg) were inoculated in MS medium supplemented with BA, KIN and TDZ at the concentrations of 0.5, 1, and 1.5 mg/l, in combination with 0.2 mg/l NAA. Along with the above-mentioned PGRs, the calli were also inoculated in PGR-free MS medium (served as the control). Data on response of calli on regeneration (%), days to fresh shoot induction were taken on a daily basis, whereas number of shoots, shoot length (mm), and number of leaves, along with fresh weight (mg) and dry weight (mg), were recorded at 56 days of culture.

### Estimation of reserpine

For estimation of the key secondary metabolite reserpine from the in vitro regenerated multiple shoots and calli, the samples were oven-dried. For the purpose of identification of reserpine and its subsequent quantification, standard reserpine (Sigma-Aldrich^®^, Bangalore, India) was used as the reference material. 1 g of hot-air oven-dried powder was considered for the quantification of the secondary metabolite for each of the samples. The samples were extracted with high-performance liquid chromatography (HPTLC)–grade methanol (via vortexing) and were then kept at room temperature (25 °C). The methanol extracted samples were then concentrated at a reduced pressure at 55 ± 2 °C in rotary evaporator. Then extracts from the samples were mixed with methanol for liquefaction to arrive at a concentration of 0.1 g/ml. Finally, these were then subjected to HPLTC analysis. After analysis, the samples were smeared over pre-coated silica gel aluminium plate 60F_254_, in form of bands with the help of Camag microlitre syringe fixed on a Camag Linomat V (Switzerland) automated sample applicator with nitrogen flow. The plates were then dried and the chromatogram lane was developed with the mobile phase comprising of toluene: ethyl acetate: diethyleamine (7:2:1; v/v/v). With the aid of Camag TLC scanner III (Switzerland), the plates were scanned at 200–400 nm after their removal from the chamber and subsequent drying. Data regarding the peak area were noted and further assessments for the reserpine content were done with Camag Win CATS software.

### Data analysis

For each of the experiments (repeated thrice) involving multiple shoot initiation, proliferation, callus induction, and callus regeneration, three replications per treatment were considered. Each of the replications consisted of ten explants. The experiments were laid out following a Completely Randomized Design. Statistical analyses of the recorded data were done employing SPSS (version 17.0, SPSS Inc., Chicago, IL, USA) software. Subsequently, one-way analysis of variance (ANOVA) was formulated and statistical significance was calculated. Furthermore, statistical assessment of the data (mean ± standard error), which differed significantly, was determined with Tukey’s test (PC version Origin 7.0 Northampton, MA, USA) at *P* = 0.05 using SPSS (Version 11, SPSS Inc. Chicago, USA) software.

## Results

### Direct regeneration: multiple shoot initiation

**Fig. 1:**
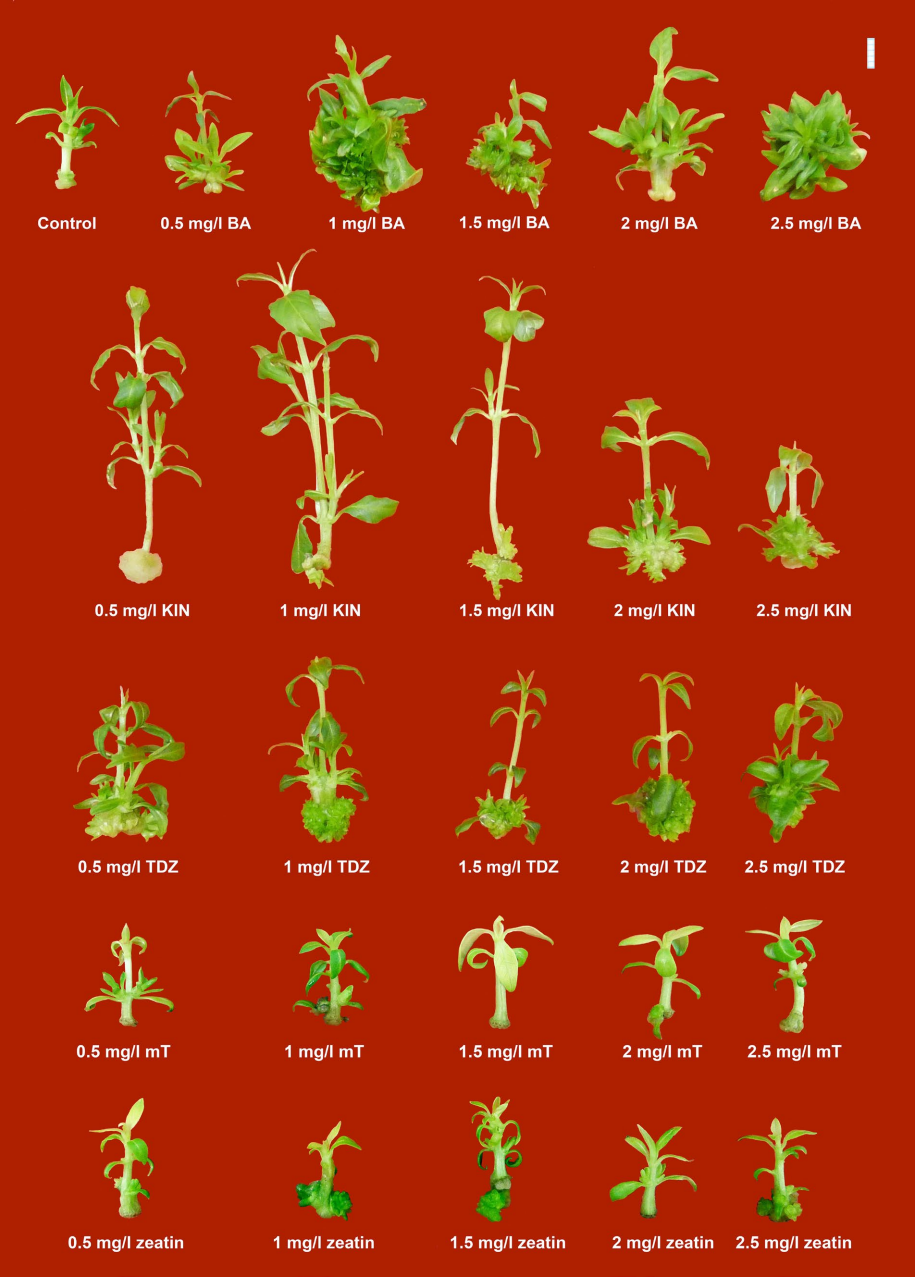
Influence of type and concentrations of plant growth regulators (cytokinins) on direct regeneration (multiple shoot initiation) in *Rauvolfia serpentina* (L.) Benth. ex Kurz. (Bar = 5 mm)

Five different cytokinins viz. BA, KIN, TDZ, zeatin, and mT were tested for their multiple shoot initiation capabilities. Maximum shoot induction was obtained in case of 1 mg/l BA that exhibited the earliest days to shoot initiation, maximum shoot multiplication, and maximum fresh and dry weights (Fig. 1). However, exceptionally, maximum response (~ 90%) and earliness to shooting were noticed even when a higher dose of BA (2.5 mg/l) was applied. As per the present study, it can be observed that the overall performances of the three PGRs viz. BA, KIN, and TDZ were much better than zeatin and mT, wherein the responses and the other growth attributes when studied showed less proliferation (Table 1). On one hand, where better multiplication rates and higher biomass production (Table 1) were observed with BA; on other hand, a better shoot elongation was observed in KIN-supplemented media formulations. Highest shoot elongation was seen in case of 1 mg/l KIN (~ 42 mm).

**Table 1:** Influence of plant growth regulators on multiple shoot initiation in *Rauvolfia serpentina* (L.) Benth. ex Kurz.

Plant growth regulators (mg/l)					Morphogenetic response						
BA	KIN	TDZ	zeatin	mT	Frequency of response to multiple shoot initiation (%)	Days to initiation of multiple shoot	No. of shoots/explant	Shoot length (mm)	No. of leaves/explant	FW (mg)	DW (mg)
0.5	–	–	–	–	83.9 ± 6.1ab	9.0 ± 1.15c–f	9.0 ± 2.3cd	15.7 ± 0.9d–f	29.0 ± 3.5a	290.0 ± 19.7c–f	27.0 ± 9.2c–e
1	–	–	–	–	68.7 ± 2.7b–f	7.3 ± 2.0f	19.0 ± 0.0a	23.0 ± 0.0b–d	19.7 ± 0.8cd	680.3 ± 131.6a	55.0 ± 11.5a
1.5	–	–	–	–	72.3 ± 8.8b–e	10.7 ± 0.3c–e	7.7 ± 1.5de	14.0 ± 0.6f	19.0 ± 0.0cd	370.0 ± 0.0b–e	30.0 ± 1.6b–e
2	–	–	–	–	72.0 ± 9.6b–e	14.7 ± 1.3b	6.3 ± 0.3ef	25.3 ± 0.3bc	22.3 ± 0.3bc	300.0 ± 40.4c–f	30.0 ± 0.6b–e
2.5	–	–	–	–	90.0 ± 0.0a	4.3 ± .3g	5.7 ± 0.3e–h	18.7 ± 2.0c–f	27.3 ± 3.2ab	420.0 ± 57.7bc	38.5 ± 0.8bc
–	0.5	–	–	–	68.8 ± 2.7b–f	11.7 ± 1.5c	6.0 ± 0.0e–g	37.0 ± 3.5a	22.3 ± 0.3bc	195.0 ± 20.2f	22.7 ± 2.6de
–	1	–	–	–	77.7 ± 6.1a–c	10.3 ± 0.3c–f	6.0 ± 0.6e–g	42.3 ± 4.3a	19.0 ± 0.0cd	230.0 ± 11.5ef	26.0 ± 3.5c–e
–	1.5	–	–	–	61.2 ± 2.2d–g	15.3 ± 0.7b	6.3 ± 0.8ef	41.7 ± 0.8a	8.7 ± 1.5h	290.0 ± 51.9c–f	27.0 ± 3.7c–e
–	2	–	–	–	68.8 ± 2.7b–f	15.0 ± 0.6b	10.7 ± 0.3bc	29.7 ± ±0.3b	20.0 ± 0.6cd	420.0 ± 69.3bc	34.0 ± 4.0b–d
–	2.5	–	–	–	77.7 ± 6.1a–c	14.7 ± 0.7b	12.0 ± 0.6b	19.0 ± 0.0c–f	16.3 ± 0.8c–f	290.0 ± 40.4c–f	20.0 ± 0.0e
–	–	0.5	–	–	77.7 ± 6.1a–c	8.0 ± 1.0d–f	7.0 ± 1.5de	25.7 ± 0.3bc	15.0 ± 0.6d–g	390.0 ± 28.9b–d	32.0 ± 1.2b–e
–	–	1	–	–	77.7 ± 6.1a–c	10.3 ± 1.2c–f	6.0 ± 0.0e–g	40.0 ± 9.8a	18.3 ± 0.3c–e	455.0 ± 66.4b	41.3 ± 4.9b
–	–	1.5	–	–	75.0 ± 7.8a–d	10.7 ± 0.3c–e	4.0 ± 0.6f–i	25.0 ± 0.6bc	15.0 ± 3.5d–g	225.0 ± 20.2ef	29.0 ± 6.4b–e
–	–	2	–	–	63.4 ± 0.0c–g	7.7 ± 0.7ef	6.0 ± 1.7e–g	22.0 ± 1.2b–e	8.0 ± 1.0h	365.0 ± 77.9b–e	30.0 ± 6.4b–e
–	–	2.5	–	–	77.7 ± 6.1a–c	9.7 ± 1.3c–f	7.7 ± 0.3de	24.0 ± 1.2bc	22.3 ± 5.5bc	260.0 ± 5.8d–f	25.3 ± 1.5d–e
–	–	–	0.5	–	50.8 ± 0.0g	8.3 ± 0.3d–f	2.3 ± 0.7ij	11.3 ± 1.3f	6.7 ± 0.3h	16.7 ± 3.3g	1.7 ± 0.7f
–	–	–	1	–	61.2 ± 2.2d–g	9.0 ± 0.6c–f	3.0 ± 0.0ij	13.0 ± 0.6f	12.7 ± 2.9e–h	30.0 ± 5.8g	3.3 ± 1.2f
–	–	–	1.5	–	57.0 ± 3.6e–g	10.0 ± 1.1c–f	3.7 ± 0.3g–i	13.7 ± 1.5f	11.0 ± 1.5f–h	30.0 ± 10.0g	3.0 ± 2.0f
–	–	–	2	–	55.0 ± 4.2 fg	10.3 ± 0.3c–f	3.3 ± 0.3h–j	13.7 ± 0.8f	9.7 ± 0.3gh	10.0 ± 0.0g	1.0 ± 0.0f
–	–	–	2.5	–	55.0 ± 4.2 fg	9.3 ± 0.3c–f	3.7 ± 0.3g–i	13.0 ± 1.0f	8.7 ± 0.8	13.3 ± 3.3g	1.0 ± 0.0f
–	–	–	–	0.5	61.2 ± 2.2d–g	8.0 ± 0.0d–f	3.0 ± 0.0ij	12.3 ± 0.8f	17.3 ± 2.2c–e	26.7 ± 3.3g	1.7 ± 0.3f
–	–	–	–	1	59.0 ± 2.2e–g	10.0 ± 1.2c–f	3.0 ± 0.0ij	12.0 ± 0.6f	14.7 ± 0.8d–g	23.3 ± 6.7g	2.0 ± 0.6f
–	–	–	–	1.5	50.8 ± 0.0g	10.3 ± 0.3c–f	1.0 ± 0.0j	15.3 ± 1.2ef	6.7 ± 0.3h	30.0 ± 5.8g	2.7 ± 0.8f
–	–	–	–	2	57.0 ± 3.6e–g	10.7 ± 0.7c–e	1.7 ± 0.3ij	13.7 ± 0.7f	8.3 ± 0.8h	18.3 ± 6.0g	1.0 ± 0.0f
–	–	–	–	2.5	54.8 ± 2.0 fg	11.0 ± 0.6cd	3.3 ± 0.3h–j	15.0 ± 0.6ef	8.7 ± 0.3h	26.7 ± 3.3g	1.7 ± 0.3f
C	–	–	–	–	48.9 ± 1.9g	18.7 ± 0.3a	3.0 ± 0.0ij	11.3 ± 0.8f	6.7 ± 0.3h	9.0 ± 1.0g	1.0 ± 0.0f

Data represent mean of three replicates with ten samples (shoot tip explants) per treatment. Growth period 42 days. (*C* Control)

Data for each column followed by the different letters are significantly different according to Tukey’s test at *P* = 0.05

Data expressed as percentage were transformed using arc sine prior to ANOVA and converted back to the original scale for demonstration in the table (Compton 1994)

### Direct regeneration: multiple shoot proliferation

**Fig. 2:**
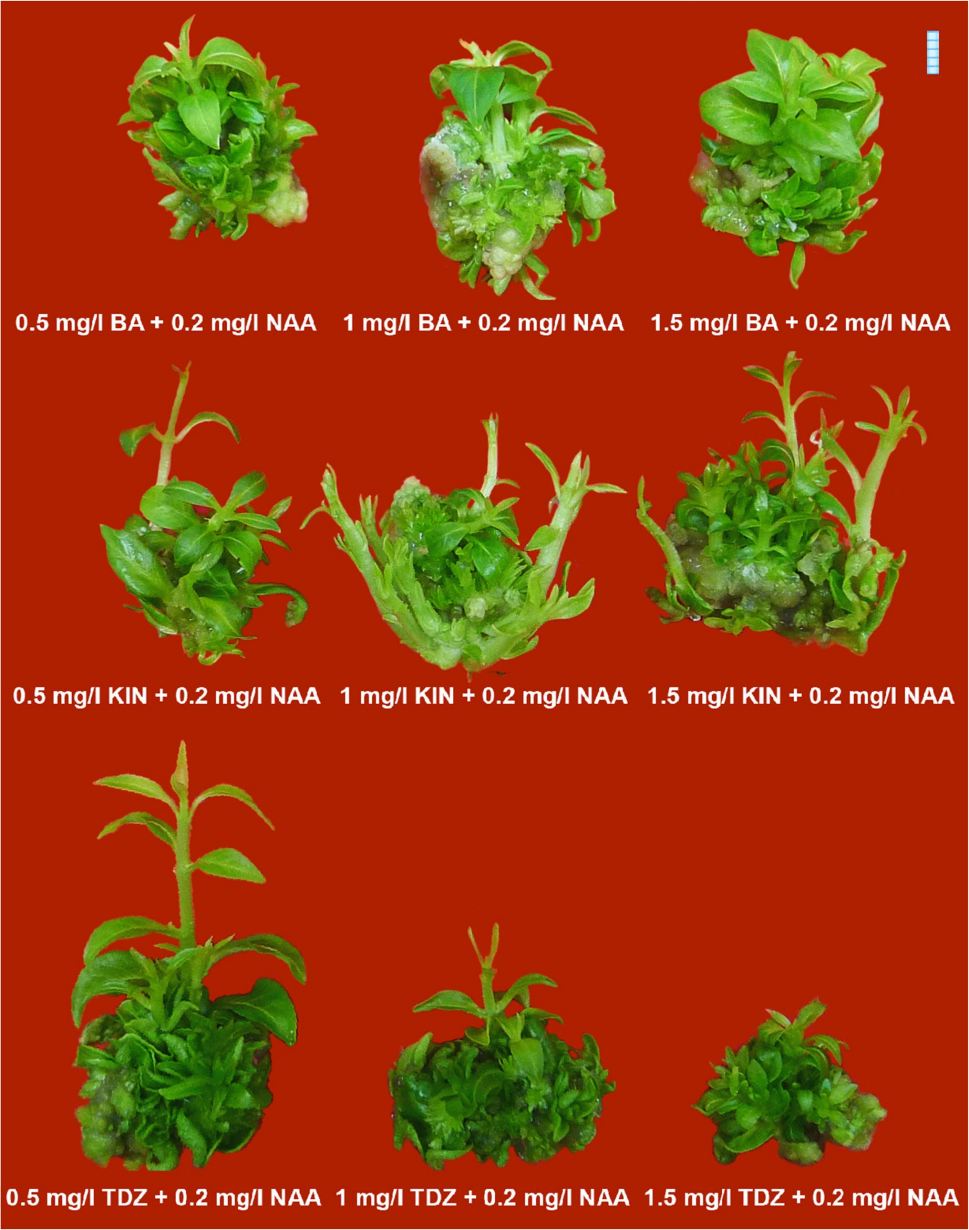
Influence of type, concentration, and combination of plant growth regulators (cytokinins:auxin) on direct regeneration (multiple shoot proliferation) in *Rauvolfia serpentina* (L.) Benth. ex Kurz. (Bar = 5 mm)

In the current experiment on multiple shoot induction, the PGRs that gave better performances were chosen for further shoot and leaf proliferation studies. The PGRs, viz., BA, KIN, and TDZ, were used in combination with 0.2 mg/l NAA to study the proliferation rates. The effects of cytokinin + auxin combinations on direct multiple shoot proliferation were studied. Maximum shoot multiplication (~ 21 shoots) and highest fresh weight (~ 1990 mg) of the proliferated mass were obtained in case of 1 mg/l KIN + 0.2 mg/l NAA (Fig. 2). Highest response percentage (~ 84%) and maximum amount of leaf proliferation was observed in case of 1.5 mg/l KIN + 0.2 mg/l NAA (~ 47 nos.), thus depicting the effectiveness of KIN in inducing higher proliferation rates. Earliest response (~ 9 days), maximum shoot elongation (~ 26 mm), and increase in biomass with respect to dry weight (~ 160 mg) were obtained in 0.5 mg/l TDZ + 0.2 mg/l NAA (Table 2). Remarkably, 0.5 BA + 0.2 NAA also exhibited the highest frequency of shoot proliferation (~ 84%). Across the different treatments, it was seen that an increase in PGR concentrations led to a decrease in proliferation in case of TDZ + NAA, however, for KIN + NAA combination an opposite trend was noticed. In case of BA + NAA combinations, intermediate concentrations exhibited better results.

**Table 2:** Influence of plant growth regulators on multiple shoot proliferation in *Rauvolfia serpentina* (L.) Benth. ex Kurz.

Plant growth regulators (mg/l)				Morphogenetic response						
NAA	BA	KIN	TDZ	Frequency of response to shoot proliferation (%)	Days to initiation of shoot proliferation	No. of shoots/explant	Shoot length (mm)	No. of leaves/explant	FW (mg)	DW (mg)
0.2	0.5	–	–	83.9 ± 6.1a	12.7 ± 0.8bc	15.7 ± 1.4b	21.0 ± 1.5ab	46.3 ± 1.8a	1280.0 ± 98.1c	90.0 ± 5.8de
0.2	1	–	–	71.6 ± 0.0b	11.0 ± 0.6b–d	14.3 ± 0.8bc	21.3 ± 3.9ab	39.3 ± 1.2b	1525.0 ± 72.1bc	121.7 ± 15.9bc
0.2	1.5	–	–	68.9 ± 2.7bc	12.0 ± 0.6b–d	15.3 ± 3.3b	21.0 ± 2.0ab	39.0 ± 1.5b	1415.0 ± 37.5c	115.0 ± 8.7cd
0.2	–	0.5	–	59.0 ± 2.2cd	12.0 ± 1.7b–d	15.3 ± 1.7b	20.7 ± 2.9ab	28.7 ± 1.7c	785.0 ± 20.2d	65.0 ± 2.9e
0.2	–	1	–	66.1 ± 2.7bc	12.7 ± 1.2bc	21.0 ± 0.6a	19.3 ± 3.8ab	34.0 ± 3.0bc	1990.0 ± 69.2a	145.0 ± 14.4ab
0.2	–	1.5	–	83.9 ± 6.1a	13.3 ± 1.5b	16.7 ± 0.8b	22.7 ± 3.7ab	47.3 ± 3.7a	1695.0 ± 112.5b	145.0 ± 2.9ab
0.2	–	–	0.5	68.9 ± 2.7bc	9.3 ± 0.9d	10.3 ± 0.8cd	26.3 ± 9.4a	37.7 ± 1.2b	1955.0 ± 31.7a	160.0 ± 5.8a
0.2	–	–	1	68.9 ± 2.7bc	10.0 ± 0.6cd	7.0 ± 1.0de	17.7 ± 2.9ab	37.7 ± 1.8b	1510.0 ± 173.2bc	137.7 ± 7.2a–c
0.2	–		1.5	66.1 ± 2.7bc	12.3 ± 0.3b–d	14.7 ± 0.3bc	15.7 ± 2.0ab	34.3 ± 0.8bc	645.0 ± 43.3d	81.7 ± 7.2e
C	–	–	–	52.8 ± 2.0d	19.7 ± 0.3a	3.0 ± 0.0e	10.3 ± 0.3b	6.7 ± 0.3d	13.3 ± 3.3e	1.3 ± 0.3f

Data represent mean of three replicates with ten samples (shoot tip explants) per treatment. Growth period 42 days (*C*  control)

Data for each column followed by the different letters are significantly different according to Tukey’s test at *P* = 0.05

Data expressed as percentage were transformed using arc sine prior to ANOVA and converted back to the original scale for demonstration in the table (Compton 1994)

### Indirect regeneration: callus induction and proliferation

**Fig. 3:**
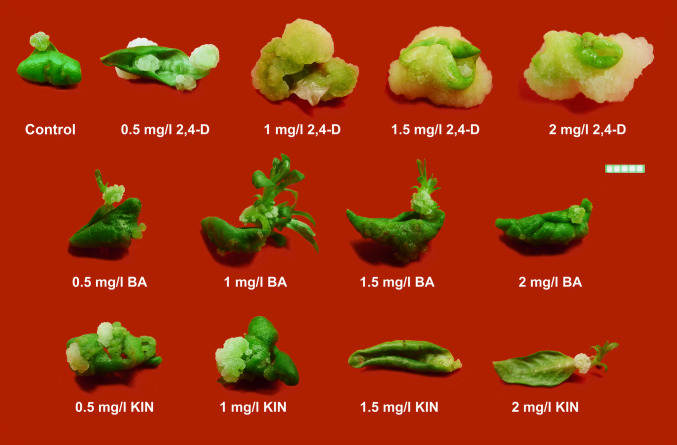
Influence of type, concentration, and combination of plant growth regulators (cytokinins and auxin) on callus induction and its proliferation from leaf explants of *Rauvolfia serpentina* (L.) Benth. ex Kurz. (Bar = 5 mm)

In most of the cases, calli were induced from the wounded portions of the leaf; in addition, induction of calli were also noticed at the leaf petiolar end (Fig. 3). The best callus induction response was obtained from 1.5 mg/l 2,4-D, wherein highest response percentages (~ 90%) and highest biomass yield with respect to fresh weight of calli (~ 830 mg) was obtained when compared to other treatments. Considering the biomass yield in terms of dry weight, 2 mg/l 2,4-D gave the best results (~ 45 mg). However, earliest callus induction was observed in 2 mg/l BA. Across the different PGR concentrations, it was noticed that an increase in concentrations of PGR led to an increase in callusing in case of 2,4-D, whereas in case of BA, callusing rates attained peak at 1 mg/l and then showed subsequent reduction. In case of KIN, even though callus induction responses fluctuated within the range ~ 45–49%, yet highest biomass yield in terms of fresh weight and dry weight were obtained from 0.5 mg/l KIN, indicating converse relation with PGR concentration (Table 3). All the calli obtained from 2,4-D were greenish white and friable, justifying their organogenic nature. The calli obtained from BA and KIN were light green in colour and compact to friable in form (Fig. 3).

**Table 3:** Influence of plant growth regulators on callus induction from leaf explants of *Rauvolfia serpentina* (L.) Benth. ex Kurz

Plant growth regulators (mg/l)			Morphogenetic response			
			Frequency of response to callus induction (%)	Days to induction of callus	FW (mg)	DW (mg)
2,4-D	BA	KIN				
0.5	–	–	50.8 ± 3.4d	13.3 ± 1.3cd	30.0 ± 10.4de	6.7 ± 1.7c
1	–	–	68.8 ± 2.7b	18.7 ± 1.5cd	285.0 ± 18.0c	26.0 ± 2.6b
1.5	–	–	90.0 ± 0.0a	20.3 ± 0.7b–d	830.0 ± 36.1a	40.7 ± 5.8a
2	–	–	90.0 ± 0.0a	26.7 ± 4.3ab	528.3 ± 64.3b	45.3 ± 8.1a
–	0.5	–	46.9 ± 1.9d	15.3 ± 1.7cd	11.7 ± 1.7e	2.0 ± 2.3c
–	1	–	59.0 ± 2.2c	18.0 ± 2.0cd	91.7 ± 9.3d	7.2 ± 0.6c
–	1.5	–	48.8 ± 1.9d	18.0 ± 2.1cd	23.3 ± 2.4e	4.2 ± 0.5c
–	2	–	46.9 ± 1.9d	13.0 ± 1.0d	13.7 ± 2.0e	2.0 ± 0.6c
–	–	0.5	48.8 ± 1.9d	20.3 ± 3.8b–d	30.7 ± 6.9de	3.9 ± 0.8c
–	–	1	46.9 ± 1.9d	21.0 ± 1.0b–d	18.7 ± 0.8e	2.1 ± 0.7c
–	–	1.5	45.0 ± 0.0d	14.3 ± 0.3cd	9.3 ± 1.2e	1.3 ± 0.3c
–	–	2	46.9 ± 1.9d	21.3 ± 3.9bc	14.3 ± 2.8e	1.3 ± 0.3c
C	–	–	21.1 ± 2.7e	33.3 ± 3.3a	13.3 ± 2.4e	1.3 ± 0.3c

Data represent mean of three replicates with ten samples (leaf explants) per treatment. Growth period 56 days (*C* control)

Data for each column followed by the different letters are significantly different according to Tukey’s test at *P* = 0.05

Data expressed as percentage were transformed using arc sine prior to ANOVA and converted back to the original scale for demonstration in the table (Compton 1994)

### Indirect regeneration: shoot regeneration from callus

**Fig. 4:**
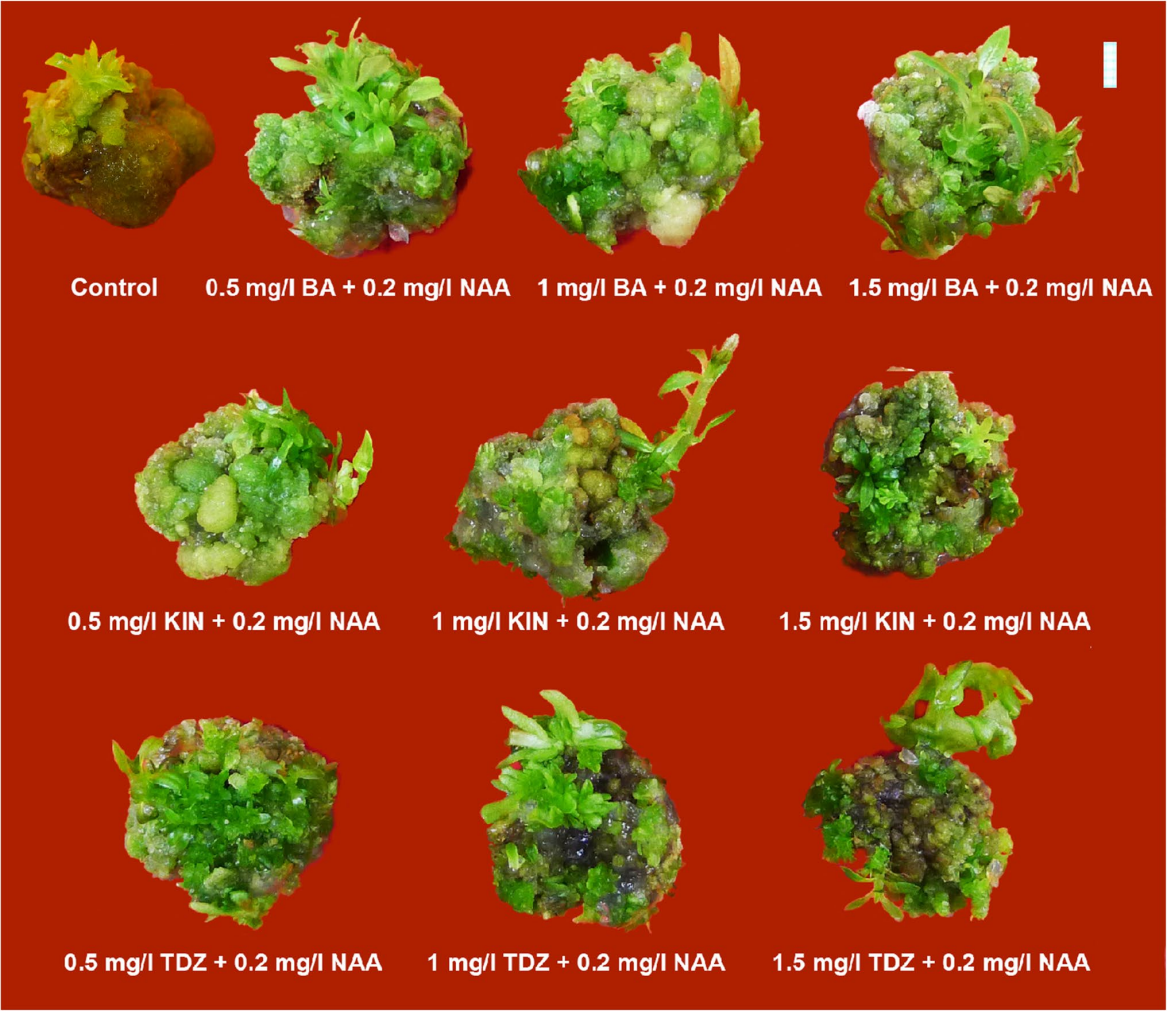
Influence of type, concentration, and combination of plant growth regulators (cytokinins:auxin) on indirect regeneration (callus-mediated regeneration of multiple shoots) in *Rauvolfia serpentina* (L.) Benth. ex Kurz. (Bar = 5 mm)

From the above-mentioned experiments on callus induction, it was found that the PGR treatments involving 1–1.5 mg/l 2,4-D were highly proliferative and organogenic, and were thus used for indirect shoot regeneration purposes. Across the different concentrations of PGRs, 1 mg/l TDZ in combination with 0.2 mg/l NAA was found to be the best in terms of shoot and leaf proliferation (Fig. 4). The highest biomass yield with respect to fresh weight (~ 3080 mg) and maximum shoot length (~ 22 mm) was observed in case of 1 mg/l KIN + 0.2 mg/l NAA when compared to other PGR combinations. However, the highest response percentage (shoot regeneration ~ 75%) and earliness to shooting (~ 3 days) were recorded in case of 1.5 mg/l KIN + 0.2 mg/l NAA. Biomass production, in terms of fresh weight and dry weights, had been impressive with 1.5 mg/l BA + 0.2 mg/l NAA combination, thereby displaying the highest dry weight of the regenerated clumps (~ 275 mg). PGR-free control medium also exhibited regeneration but with the least frequency. Proliferation rates showed converse responses with PGR concentrations in case of BA + KIN combinations, however, in case TDZ + NAA combinations, intermediate PGR level gave better response (Table 4). Hence, KIN at an intermediate dose proved to be an effective PGR for the purpose of shoot elongation in the present study.

**Table 4:** Influence of plant growth regulators on indirect shoot regeneration from callus of *Rauvolfia serpentina* (L.) Benth. ex Kurz

Plant growth regulators (mg/l)				Morphogenetic response						
NAA	BA	KIN	TDZ	Frequency of response to shoot initiation (%)	Days to initiation of shoot initiation	No. of shoots/explant	Shoot length (mm)	No. of leaves/explant	FW (mg)	DW (mg)
0.2	0.5	–	–	70.1 ± 10.1a	6.7 ± 0.88bc	13.0 ± 4.0a	13.3 ± 0.8b	27.0 ± 2.8a	2625.0 ± 233.8ab	240.0 ± 11.5a
0.2	1	–	–	61.2 ± 2.2a	3.7 ± 0.6c	9.3 ± 1.5ab	18.3 ± 0.3a	22.7 ± 5.5a	2975.0 ± 95.2ab	265.0 ± 20.2a
0.2	1.5	–	–	61.2 ± 2.2a	5.7 ± 1.8bc	8.7 ± 0.8ab	13.0 ± 2.5b	27.7 ± 5.8a	2920.0 ± 115.5ab	275.0 ± 14.4a
0.2	–	0.5	–	72.3 ± 8.9a	5.0 ± 1.5bc	15.0 ± 4.0a	13.3 ± 0.8b	26.7 ± 5.2a	2885.0 ± 453.2ab	255.0 ± 20.2a
0.2	–	1	–	63.9 ± 4.3a	8.7 ± 0.7b	11.3 ± 1.8ab	22.0 ± 1.7a	25.3 ± 7.2a	3080.0 ± 103.9a	235.0 ± 8.7a
0.2	–	1.5	–	75.0 ± 7.8a	3.3 ± 0.3c	14.3 ± 2.0a	11.0 ± 0.6b	17.0 ± 2.1ab	2285.0 ± 222.3ab	265.0 ± 8.6a
0.2	–	–	0.5	68.8 ± 13.1a	5.3 ± 1.2bc	12.3 ± 4.9a	12.0 ± 1.7b	30.0 ± 8.7a	2795.0 ± 522.5ab	245.0 ± 31.7a
0.2	–	–	1	72.3 ± 8.8a	4.0 ± 0.6c	16.7 ± 2.0a	18.3 ± 0.8a	32.3 ± 4.1a	2115.0 ± 210.7b	245.0 ± 31.7a
0.2	–		1.5	59.0 ± 2.2a	5.7 ± 0.7bc	8.3 ± 0.8ab	12.3 ± 1.5b	18.3 ± 1.5ab	2460.0 ± 40.4ab	225.0 ± 2.8a
C	–	–	–	21.1 ± 2.7b	14.3 ± 1.7a	2.7 ± 0.3b	6.3 ± 0.8c	3.7 ± 0.3b	362.3 ± 51.9c	42.0 ± 5.1b

Data represent mean of three replicates with ten samples (callus explants) per treatment.*Growth period 56 days (*C* control)

Data for each column followed by the different letters are significantly different according to Tukey’s test at *P* = 0.05

Data expressed as percentage were transformed using arc sine prior to ANOVA and converted back to the original scale for demonstration in the table (Compton 1994)

### Quantification of reserpine level

**Fig. 5:**
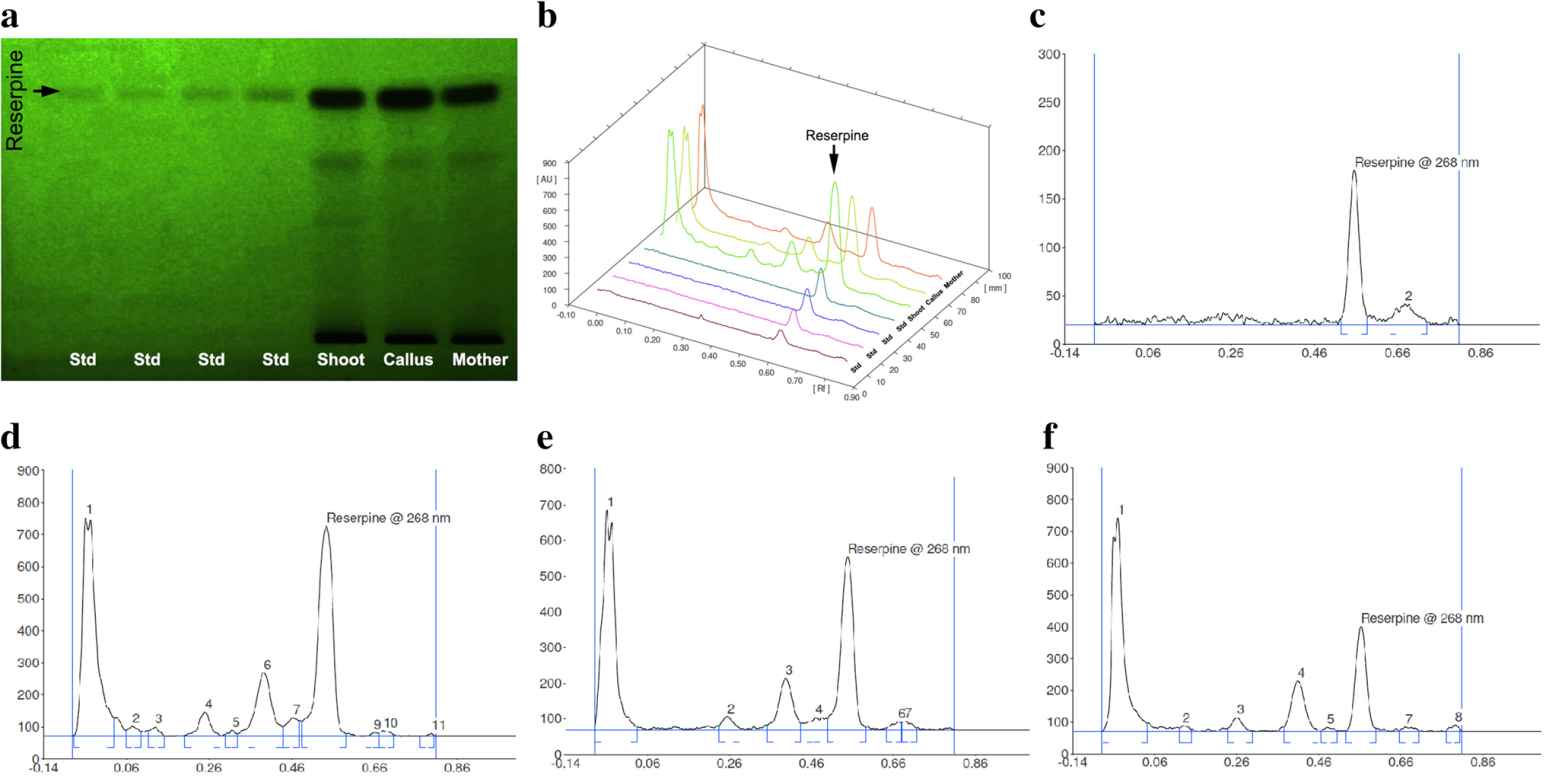
Estimation of reserpine content in the extract from in vitro shoots (direct regeneration), calli (indirect regeneration), and shoots of mother plant of *Rauvolfia serpentina* (L.) Benth. ex Kurz. **a** HPTLC fingerprint of the extracts; **b** HPTLC 3D overlay densitogram of reserpine standard with that of the extracts obtained from in vitro shoots, calli and shoots of mother plant; chromatographs of **c** reserpine standard, **d** extract from in vitro shoot, **e** calli, and **f** mother plant (shoot)

During the conduction of the present experiment, the reserpine standard displayed a solitary peak in the HPLTC chromatogram. The maximum value of UV for the estimation of reserpine from the samples of *R. serpentina*, consisting of multiple shoots from direct regeneration, callus-mediated regeneration, and the mother plant, was selected with help of the UV spectrum of reserpine obtained before and after completion of the experiment. The chromatogram obtained from the standard reserpine exhibited the peak at 268 nm (Fig. 5). Calculation of the peak area from the obtained graph exhibited that in vitro shoots yielded 503 μg/g of reserpine on dry weight basis, whereas in vitro callus yielded 319 μg/g of reserpine. Shoots from mother plant yielded 184 μg/g of reserpine on dry weight basis. Thus, an enhancement in the reserpine content under in vitro culture via both direct shoot regeneration systems (~ three folds) and indirect (callus-mediated) regeneration systems (~ two folds) was achieved in the present study following this refined protocol.

## Discussion

For in vitro multiple shoot initiation in *R. serpentina* in the present study, BA outperformed the other four cytokinins (namely, KIN, TDZ, zeatin and mT). Presumably, BA is effective in breaking apical dominance, thereby promoting more lateral bud initiation and subsequent growth (also confirmed by Bahuguna et al. 2011). Such result justifies that this type and dose of PGR is optimum for the direct growth and development of meristematic tissues. Initiation and multiplication rates, including biomass enhancement, were much superior in case of BA than the other PGR types and concentrations, which was also established in the reports of Mishra et al. (2010), Alatar et al. (2012), George et al. (2012), and Faisal et al. (2012) in *Rauvolfia* spp. Irrespective of PGR type, lower concentrations exhibited better performance than the higher ones, with the exception of BA. Similar results were published in the reports of Mallick et al. (2012) and Zafar et al. (2019) wherein it was claimed that shoots were more responsive to higher concentrations of BA during shoot induction. Most likely, a higher dose of BA was effective in shoot initiation and lower concentrations of the same induced better shoot growth and proliferation. In *Rauvolfia*, across several published reports, it was detected that the effects of zeatin and mT on shoot multiplication were not explored (reviewed by Mukherjee et al. 2019) till date, which has been duly addressed in the present study. It was further noticed that application of BA resulted in enhanced shoot multiplication and higher biomass content, whereas application of KIN resulted in elongation of shoots. Such findings are in homology with the reports of Shivaraj and Rao (2011) in eggplant (*Solanum melongena* L.), wherein the effectiveness of BA (in obtaining higher frequency of shooting) and KIN (in shoot elongation) were highlighted. In most of the published reports related to the direct regeneration (i.e., direct multiple shoot proliferation) in *Rauvolfia*, the effects of combination media, viz., BA + KIN + IAA or NAA, have been studied. During proliferation of initiated multiple shoots, TDZ in combination of NAA significantly outperformed the other cytokinins (BA or KIN). Such beneficial effects of TDZ on inducing shoot multiplication and elongation were also reported by Alatar (2015). As per the reports of Mondal et al. (2011), Alatar et al. (2012), Rani et al. (2014), and Ahmad et al. (2015), it was confirmed that media containing cytokinin + auxin combinations were effective in direct shoot regeneration. However, contrary to their reports, wherein it was claimed that BA + NAA combinations were much more effective in shoot proliferation; the present experiment has revealed, otherwise, that KIN + NAA combinations yielded better results.

Leaf explants chosen for callus induction were subjected to different PGRs, which exhibited differential rates of callus induction and proliferation. Efficiency of leaf explants during induction of callus in *R. serpentina* was reported by majority of the earlier studies (reviewed by Mukherjee et al. 2019). Calli induced with the application of 2,4-D were organogenic and greenish white. Similar findings were reported by Bahuguna et al. (2011), Mallick et al. (2012), Rohela et al. (2013), and Gantait et al. (2017). On the other hand, BA- or KIN-induced calli were light green and compact to friable in nature. This is in accordance with the results that were reported by Singh et al. (2009) and Rohela et al. (2013) on *R. serpentina*. Contrary to our results, whitish calli from BA + IAA were obtained by Rani et al. (2014) and friable calli from BA alone were reported by Zafar et al. (2019). In comparison to control, wherein minute callus was observed to have formed from the petiolar end, it could be clearly stated that in PGR-supplemented media, especially auxins like 2,4-D were utilized for inducing friable calli, and cytokinins (BA and KIN) employed for inducing green embryogenic calli, proved to be much more efficient. The above findings are in conformity with the reports of Mallick et al. (2012, 2013), and Rashmi and Trivedi (2016) on *R. serpentina*, where solely 2,4-D was responsible for callus formation, although the dose of PGR had been higher (2.5 mg/l) than the present report. However, Rashmi and Trivedi (2016) further reported the use of BA + 2,4-D combination for callus induction. In the reports of Pandey et al. (2007), Bahuguna et al. (2011), Panwar et al. (2011), Rohela et al. (2013), and Zafar et al. (2019), it was stated that 1–2 mg/l 2,4-D along NAA or BA or KIN was effective in higher callus inductions. However, Gantait et al. (2017) reported the use of a higher dose of 2,4-D (5 mg/l) along with 2 mg/l NAA for callus induction in *R. serpentina*. Contrary to the present study, Singh et al. (2009), Saravanan et al. (2011), Rani et al. (2014), Kaur (2018), and Pant and Joshi (2018) had obtained maximum callus induction using PGRs like BA, KIN, NAA, IBA, and IAA, instead of using 2,4-D.

Interaction between cytokinins and auxins in shoot regeneration from callus cultures was explored in the present study. Shoot regeneration from calli, employing TDZ alone, has also been reported by Pandey et al. (2007), although a dose that was as high as 4 mg/l was used. Reports of Rohela et al. (2013) also highlighted the use of TDZ (2.27 µM) for successful shoot regeneration. In the same way, Kaur (2018) also reported high callus induction using solely BA or KIN. Contrary to the result of the present study, in most of the other reports related to shoot regeneration from calli, BA + NAA combinations were frequently used (Panwar et al. 2011, Mallick et al. 2012, Rashmi and Trivedi 2016). However, Uikey et al. (2016) mentioned the usage of three PGRs interventions such as BA, TDZ, and NAA (each at a dose of 0.5 mg/l) for shoot regeneration from embryogenic callus-derived somatic embryos. In some cases, indirect (callus-mediated) shoot regeneration was obtained in the media supplemented with GA_3_ along with the presence of BA (Singh et al. 2009; Bahuguna et al. 2011; Gantait et al. 2017). However, unlike the present experiment, not a single study (related to callus regeneration in *Rauvolfia*) reported the use of KIN + NAA and TDZ + NAA combinations, solely (reviewed by Mukherjee et al. 2019). In majority of the reports, KIN was used in combination with BA, NAA, or IAA individually (Rohela et al. 2013; Rani et al. 2014).

Based on the HPTLC analysis of the samples, multiple shoots (via direct regeneration) exhibited higher reserpine content than calli. Possible explanation to this could be that the cells and tissues under direct regeneration have a definite organization, so the cells are oriented towards a defined biological development, which leads to a uniform biosynthesis of secondary metabolites. On the other hand, calli have an unorganized constitution, wherein not all cells might be active and hence are oriented towards a defined development and secondary metabolite production. The obtained result (reserpine content of the mother plant) of the present experiment corresponds with the reports of Panigrahi et al. (2017), wherein it was mentioned that in vitro cultured plant parts contain significantly higher secondary metabolites than that of the same ex vitro plant parts. For instance, a lower value of reserpine from ex vitro leaves (13.77 μg) was reported by Panda et al. (2010). Contrary to our results, much higher values of secondary metabolite content were reported in the findings of Panwar and Guru (2011), where the crude alkaloid contents in various parts of in vitro regenerants through HPTLC were quantified. It was mentioned in their report that in vitro leaves yielded 2.35 mg/g reserpine and stem and leaf-derived calli yielded 6.81 mg/g and 8.98 mg/g reserpine, respectively. In addition, the afore-mentioned report stated the richness of reserpine content in de-differentiated calli than well-organized shoot and leaf structures, which differs from the findings of the present study. As per the report of Panwar and Guru (2015), it was stated that in vitro regenerants produced 55.67 mg/g reserpine on dry weight basis. However, such enhancement was obtained only through salicylic acid and tryptamine elicitations. Similar usage of elicitors for reserpine enhancement was reported by Nurcahyani et al. (2008), Harisaranraj et al. (2009), Zafar et al. (2017); thus, differing from the results of the present experiment, wherein enhancement of the secondary metabolite was affected without the use of any elicitor. There are multiple reports related to reserpine estimation from in vitro shoot or calli or cell suspension culture or synthetic seeds or hairy roots individually (reviewed by Mukherjee et al. 2019). Investigations of Gantait and Kundu (2017) reported 249.37 ± 0.21 µg/gm reserpine content from germinated synthetic seeds that were kept under storage at 8 °C, whereas Faisal et al. (2013) reported a homogeneity in reserpine levels between the germinated synthetic seeds (that were stored under 4 °C storage) and ex vitro grown mother plant. Nonetheless, the present report is the first one of its kind that provides a comparative analysis of reserpine production between mother plants and regenerants from in vitro cultures, raised via direct (shoot multiplication) and indirect (callus-mediated) pathways.

## Conclusion

In the present study, a comprehensive regeneration protocol using both direct (multiple shoot) and indirect (callus-mediated) systems, under in vitro condition, has been developed. This is the only report wherein a comparative study regarding shoot initiation and its subsequent proliferation under the influence of five different PGRs viz. BA, KIN, TDZ, zeatin, and mT have been studied. Additionally, contrary to most other reports, wherein either BA + NAA or KIN + NAA combinations had been used to study shoot proliferation and/or indirect shoot regeneration from calli; the present report explores the influence of the above-mentioned PGR combinations along with TDZ + NAA to further bring about a significant enhancement in shoot multiplications and indirect shoot regenerations from calli. Similarly, the PGR type and concentration for better callus induction, using leaf explants, has also been studied in this experiment. This report exclusively provides an estimation of the enhanced (two–three folds) reserpine content from in vitro shoot and callus cultures in comparison to the ex vitro grown mother plant. Thus, in vitro direct regeneration system proved to be a more effective and efficient approach in ameliorating reserpine content.
